# Retained Digital Flexible Ureteroscopes

**DOI:** 10.1089/cren.2017.0003

**Published:** 2017-02-01

**Authors:** Melissa Huynh, Siobhan Telfer, Stephen Pautler, John Denstedt, Hassan Razvi

**Affiliations:** Division of Urology, Department of Surgery, Western University, London, Canada.

**Keywords:** retained ureteroscope, ureteroscopy, complications

## Abstract

This report documents two instances of retained flexible ureteroscopes at the time of ureteroscopy and laser lithotripsy in a healthy 37-year-old male and a 53-year-old male with a pelvic kidney. We describe maneuvers to remove the ureteroscope endoscopically in the first case, while the second case required conversion to open surgery for ureteroscope extrication.

## Case 1

### Clinical history

A 37-year-old male with recurrent nephrolithiasis underwent flexible ureteroscopy with the digital Olympus URF-V2 (Olympus Corporation, Center Valley, PA) and holmium:YAG laser lithotripsy for a 10 mm proximal right ureteral stone at a community hospital.

There were no difficulties inserting the ureteroscope, which was advanced over a guidewire without the need for ureteral balloon dilation. Upon completion of laser lithotripsy, the ureteroscope would not withdraw. The instrument was left in place for 48 hours to allow passive ureteral dilation to facilitate its removal. After another unsuccessful attempt at scope extraction, the patient was transferred to our academic center for further management.

### Physical exam

The patient was afebrile with normal vital signs. Examination revealed minimal discomfort, a Foley catheter in the bladder, the flexible ureteroscope *in situ*, and a guidewire within the working channel of the scope.

### Diagnosis

**FIG. 1.**
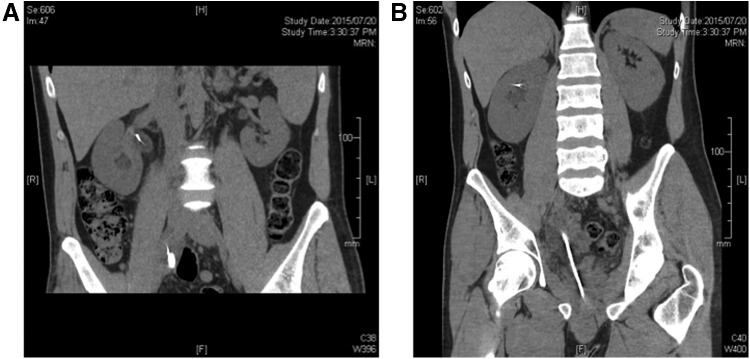
**(A, B)** CT demonstrating the wider proximal tip of the scope and the rippling appearance of the shaft.

CT kidney, ureter, and bladder radiograph (KUB) revealed mild right hydroureteronephrosis with the proximal tip of the ureteroscope at the level of the iliac vessels and a guidewire extending into an upper pole calix. Presacral and periureteric stranding and fluid tracking suggested possible ureteral perforation. No sizeable stone fragments were identified. Ripples in the outer skin of the ureteroscope were noted (Fig. 1A, B).

To exclude ureteral perforation, retrograde pyelography was performed through the working channel of the ureteroscope, demonstrating no contrast extravasation.

### Intervention

Endourologic versus open surgical interventions were discussed, and the patient agreed to an endourologic approach.

Under general anesthesia, a gentle attempt to extract the scope over a Teflon-coated guidewire was met with resistance. A hydrophilic guidewire was advanced alongside the ureteroscope until resistance was met about 2 cm from the tip of the ureteroscope. Under fluoroscopy, folds in the ureteroscope seemed to extend from the point of resistance to its tip, producing an accordion-like distortion. We bypassed the area of resistance by buttressing the wire using a 5F open-ended catheter. The open-ended catheter itself would not advance proximal to the accordioned segment and was therefore exchanged for a Kumpe catheter (Cook Urological, Spencer, IN), which then easily advanced over the wire and into the kidney. Retrograde pyelography through the Kumpe catheter redemonstrated hydroureteronephrosis, but no extravasation. A Teflon-coated wire was reinserted, and traction on the scope was again met with resistance. Orthopedic bolt cutters were used to transect the body of the scope near the hand piece, with the rationale that it might allow advancement of a ureteral access sheath over the shaft to eliminate the accordioning effect.

To minimize ureteral trauma from the passage of an access sheath, an Amplatz 8–10F coaxial dilator was advanced first over the guidewire, followed by a 12–14F ureteral access sheath over the ureteroscope. Resistance was met unexpectedly at the intramural ureter, well away from the initial deformity. Direct visualization was sought by advancing a 9.5F semirigid ureteroscope through the intramural ureter. No stones were identified, but additional folds of the ureteroscope appeared to bind it proximal to the orifice. The Amplatz 8–10 coaxial dilator was passed again to provide additional dilation which, upon gentle traction, released the ureteroscope and facilitated its removal.

Retrograde pyelography confirmed ureteral continuity, and a Double-J stent was placed. The ureteroscope was inspected and appeared intact. Cystoscopy demonstrated no foreign bodies within the bladder or the ureteral orifice.

### Follow up

CT KUB on postoperative day 1 confirmed appropriate stent position, with no retained fragments. Antibiotics were continued for 2 weeks, and the stent was removed 6 weeks postoperatively. Intravenous pyelography at 3 months demonstrated normal contrast excretion bilaterally with no evidence of hydronephrosis, stricture formation, or ureteral deformity.

### Outcomes

The patient suffered no additional complications and has not required further treatment for urolithiasis. This case highlights effective endourologic management of a retained ureteroscope.

## Case 2

### Clinical history

**FIG. 2.**
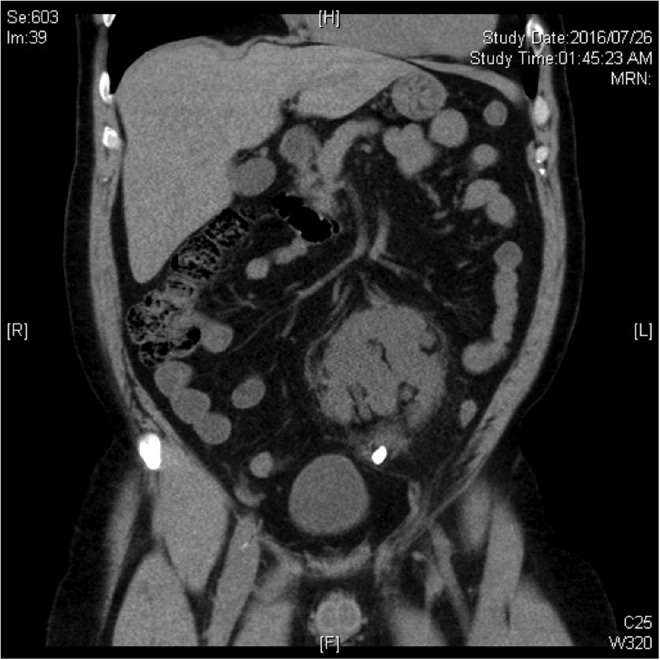
Coronal section of a noncontrast CT scan demonstrating a 1.1 cm proximal obstructing ureteric stone in a left pelvic kidney.

A 53-year-old male presented with left lower quadrant pain and intractable nausea and vomiting. He was known to have a left pelvic kidney, previous open nephrolithotomy for a right staghorn stone, and extracorporeal shockwave lithotripsy (SWL) for renal stones a number of years ago. A CT scan now demonstrated a 1.1 cm proximal ureteric stone in the pelvic kidney, with associated hydroureteronephrosis (Fig. 2). Given his elevated white count and creatinine and risk of sepsis, a Double-J stent was urgently placed without difficulty. The patient was subsequently consented for flexible ureteroscopy and laser lithotripsy for definitive stone treatment 7 weeks later.

### Diagnosis

**FIG. 3.**
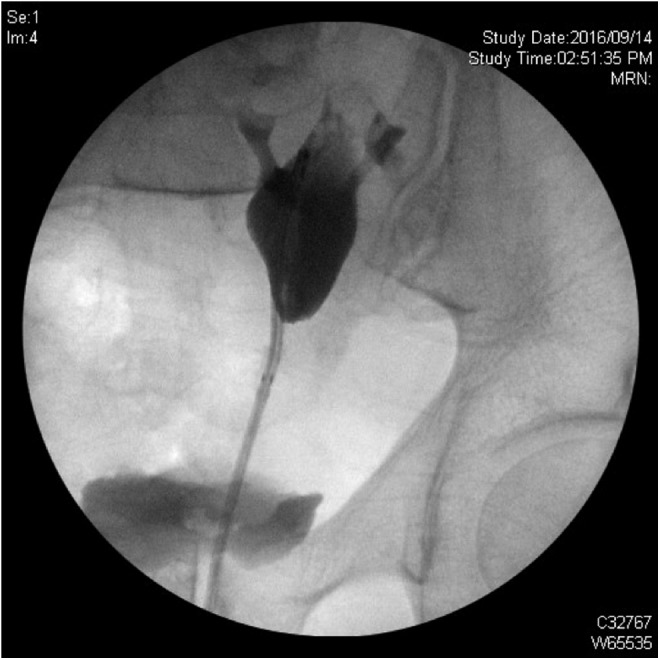
Intraoperative fluoroscopic image of contrast injection through the retained ureteroscope.

Intraoperatively, there was some difficulty in rewiring and removing the stent that was *in situ*, which seemed to catch at the level of the stone. It was eventually extracted, and the digital flexible ureteroscope (Olympus Corporation, Center Valley, PA) was advanced over a guidewire to the level of the stone, while another safety wire remained in place. During laser lithotripsy with a 270 μm holmium:YAG laser fiber, several fragments migrated proximally, therefore, the ureteroscope was advanced into the renal pelvis. Once satisfied with our stone fragmentation, we attempted to extract the ureteroscope, but it would not withdraw. Contrast was injected through the ureteroscope, and on fluoroscopy the entire kidney appeared to move with attempts to pull back on the scope (Fig. 3). An angled catheter was passed over the remaining guidewire as far proximally as possible. Contrast was injected, and none was seen to pass beyond the area where the ureteroscope was fixed.

### Intervention

To preserve the ureteroscope, a ureteral access sheath was cut along its length and passed over the ureteroscope. Unfortunately this was unsuccessful, as were attempts to replace guidewires alongside the ureteroscope. Ultimately, the ureteroscope was transected with a bolt cutter to allow further manipulation with an intact ureteral access sheath. This released the outer sheath of the ureteroscope, but the wires of the deflection mechanism and fiber optics remained fixed inside. After discussion with the patient's family, the procedure was converted to an open surgical operation.

**FIG. 4.**
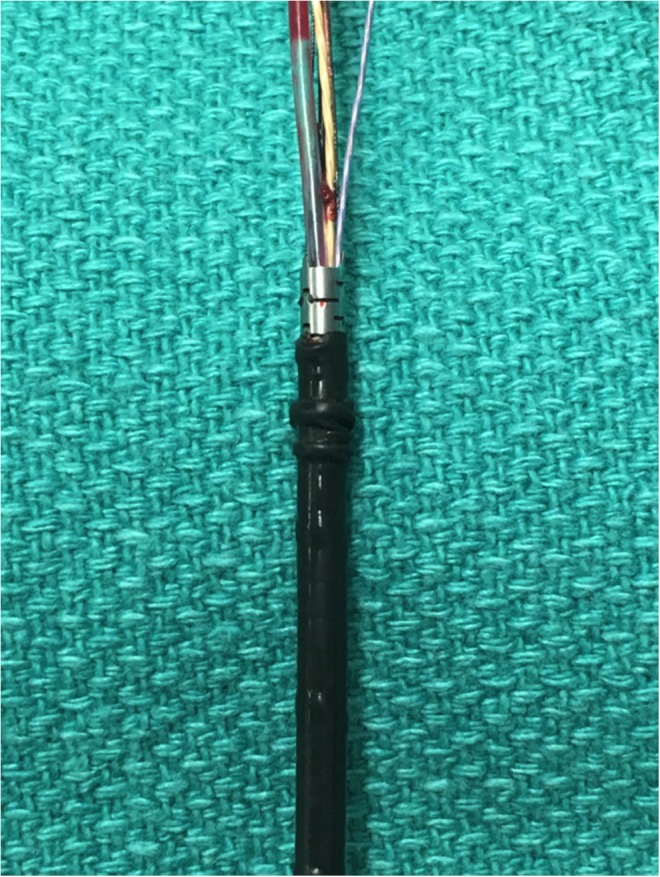
Accordioning of the distal bending rubber component of the retained ureteroscope.

To access the pelvic kidney, a midline infraumbilical incision was made and the retropubic space was developed, remaining entirely extraperitoneal. The ureter and retained ureteroscope were identified by palpation, traced to the renal pelvis, and an anterior pyelotomy was made. The ureteroscope was visualized, grasped, and removed with all components intact, along with several stone fragments. Inspection of the ureteroscope revealed accordioning of the distal bending rubber component (Fig. 4), likely against a stone fragment, rendering it immobile within the ureter. A Double-J stent was placed in an antegrade manner. Cystoscopy verified the presence of the distal curl in the bladder. The anterior pyelotomy and skin incision were closed, with a drain and urethral catheter left *in situ*.

### Follow-up

A postoperative KUB did not identify any obvious residual stone or ureteroscope fragments. The patient was discharged on postoperative day 4 following drain removal. The patient's urethral catheter and stent were removed 1 and 4 weeks postoperatively, respectively.

### Outcomes

The patient recovered well and is currently stone-free. This case underlines several endoscopic maneuvers attempted to retrieve a retained ureteroscope before resorting to an open surgical intervention for extraction.

## Discussion

Flexible ureteroscopy has an important role in the management of upper urinary tract stone disease. In many jurisdictions, it has supplanted SWL as first-line treatment for stones <2 cm given higher stone-free rates, quicker stone clearance, and a comparable safety profile.^1,2^

No previous reports of retained flexible ureteroscopes were identified in our literature review. Both of our cases appeared to result from accordioning of the outer scope shaft skin. Whether a preexisting defect in the scope or some event during the case resulted in the deformity is unclear. Stone fragments lodged alongside the ureteroscope could potentially increase the risk of this rippling deformity.

A retrospective analysis of Olympus flexible ureteroscope repairs revealed that the most frequently damaged component of the ureteroscope is the same outer bending rubber of the deflection mechanism.^3^ Tracking information for our second case revealed that the scope had been used 82 times for a total of 2010 minutes. The ureteroscope from the first case did not have tracking information available, as it originated from a peripheral center.

Olympus America Inc, recently released a Medical Device Safety Notice issuing recommendations to inspect all bending sections preoperatively and to refrain from ureteroscope usage if resistance is appreciated on insertion.^4^

## Conclusions

We document two cases of retained flexible ureteroscopes. Endourologic management was effective in one, with no further sequelae. The second patient, however, required open surgery for ureteroscope removal, as endoscopic maneuvers were ineffective.

It is unclear what led to the development of the rippling of the outer distal skin of the scopes. Whether this shearing resulted from an intrinsic manufacturing defect, the sterilization process, or wear and tear remains to be determined.

While the entrapped ureteroscopes resulted in morbidity for these patients, such complications are exceedingly rare, and flexible ureteroscopy remains an important intervention in the management of urolithiasis.

## Abbreviations Used

CT: computed tomography
IVP: intravenous pyelography
KUB: kidney, ureter, and bladder radiograph
SWL: extracorporeal shockwave lithotripsy
